# Reframing the clinical phenotype and management of cryptococcal meningitis

**DOI:** 10.1136/pn-2024-004133

**Published:** 2024-07-12

**Authors:** Maria Francisca Rocha, Hamish D C Bain, Neil Stone, David Meya, Lucia Darie, Ahmed K Toma, Michael P T Lunn, Arpan R Mehta, Charles Coughlan

**Affiliations:** 1 National Hospital for Neurology and Neurosurgery, Queen Square, University College London Hospitals NHS Foundation Trust, London, UK; 2 Hospital for Tropical Diseases, University College London Hospitals NHS Foundation Trust, London, UK; 3 Infectious Diseases Institute, Makerere University, Kampala, Uganda; 4 Department of Neurosurgery, National Hospital for Neurology and Neurosurgery, Queen Square, University College London Hospitals NHS Foundation Trust, London, UK; 5 MRC Protein Phosphorylation & Ubiquitylation Unit, School of Life Sciences, University of Dundee, Dundee, UK

**Keywords:** NEUROSURGERY, INFECTIOUS DISEASES, TROPICAL NEUROLOGY, AIDS

## Abstract

Cryptococcal meningitis is an important global health problem, resulting from infection with the yeast *Cryptococcus*, especially *Cryptococcus neoformans* and *Cryptococcus gattii*, which cause a spectrum of disease ranging from pulmonary and skin lesions to life-threatening central nervous system involvement. The diagnosis and management of cryptococcal meningitis have substantially changed in recent years. Cryptococcal meningitis often occurs in people living with advanced HIV infection, though in high-income countries with robust HIV detection and treatment programmes, it increasingly occurs in other groups, notably solid-organ transplant recipients, other immunosuppressed patients and even immunocompetent hosts. This review outlines the clinical presentation, management and prognosis of cryptococcal meningitis, including its salient differences in people living with HIV compared with HIV-negative patients. We discuss the importance of managing raised intracranial pressure and highlight the advantages of improved multidisciplinary team working involving neurologists, infectious disease specialists and neurosurgeons.

## Introduction

**Figure 1:**
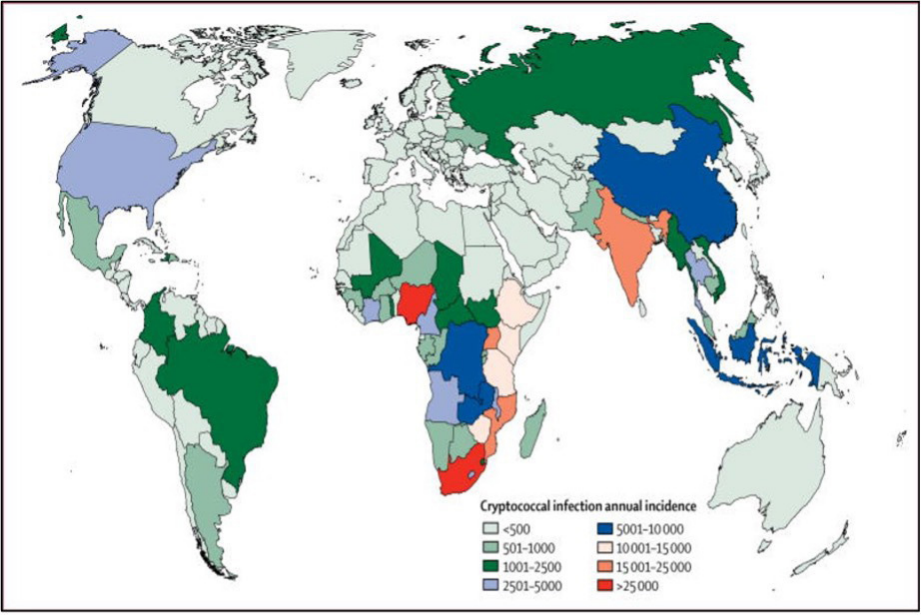
Global epidemiology of cryptococcal meningitis (CM). Map depicting annual incidence of cryptococcal meningitis, per 100 000 people, as of 2014. The burden of cryptococcal meningitis and CM-related mortality are unequally distributed worldwide. As of 2014, approximately 73% of cases occurred in sub-Saharan Africa, 22% in the Asia/Pacific region, and 1% in Europe, Latin and North America. 12-month case fatality rates range from 70% in low-income countries to 40% in middle-income countries, and 30% and 20% in Europe and North America, respectively. Reprinted from The Lancet Infectious Diseases, Vol. number 17(8) Rajasingham *et al*, Global burden of disease of HIV-associated cryptococcal meningitis: an updated analysis. 873–881.^2^ Copyright (2017), with permission from Elsevier.

 Advanced HIV infection is the cardinal risk factor for cryptococcal meningitis (CM). Since the advent of cheap, widely available antiretroviral therapy, the incidence of CM has diverged in high-income, middle-income and low-income countries (figure 1). Despite a modest fall in CM cases in low-income and middle-income countries between 2014 and 2020, it still accounts for 19% of global mortality among people living with HIV.^1^ This probably reflects the challenges of HIV and cryptococcal antigenaemia detection, chronic disease management and long-term concordance with antiretroviral therapy. CM may, therefore, be considered a ‘canary in the coalmine’ for shortcomings in HIV programmes, often representing the final event in a cascade of failed HIV care.^2 3^ In high-income countries, HIV-negative patients comprise up to 60% of CM cases. There is a growing recognition that cryptococcosis can also arise in immunocompetent people, notably within East Asian populations.^4^ It is difficult to estimate accurately CM incidence in the UK since these data are not routinely collected. A modelling study using demographic data from 2011 estimated that there were around 100 cases per year.^5^ However, this may be an underestimate as the proportion of immunosuppressed patients has increased significantly since 2011, and cases may go unrecognised in HIV-negative and immunocompetent hosts. Clinicians should consider CM in HIV-negative populations, and neurologists working in specialist infectious disease, haematology and organ transplantation centres should be particularly alert to this problem.

**Figure 2:**
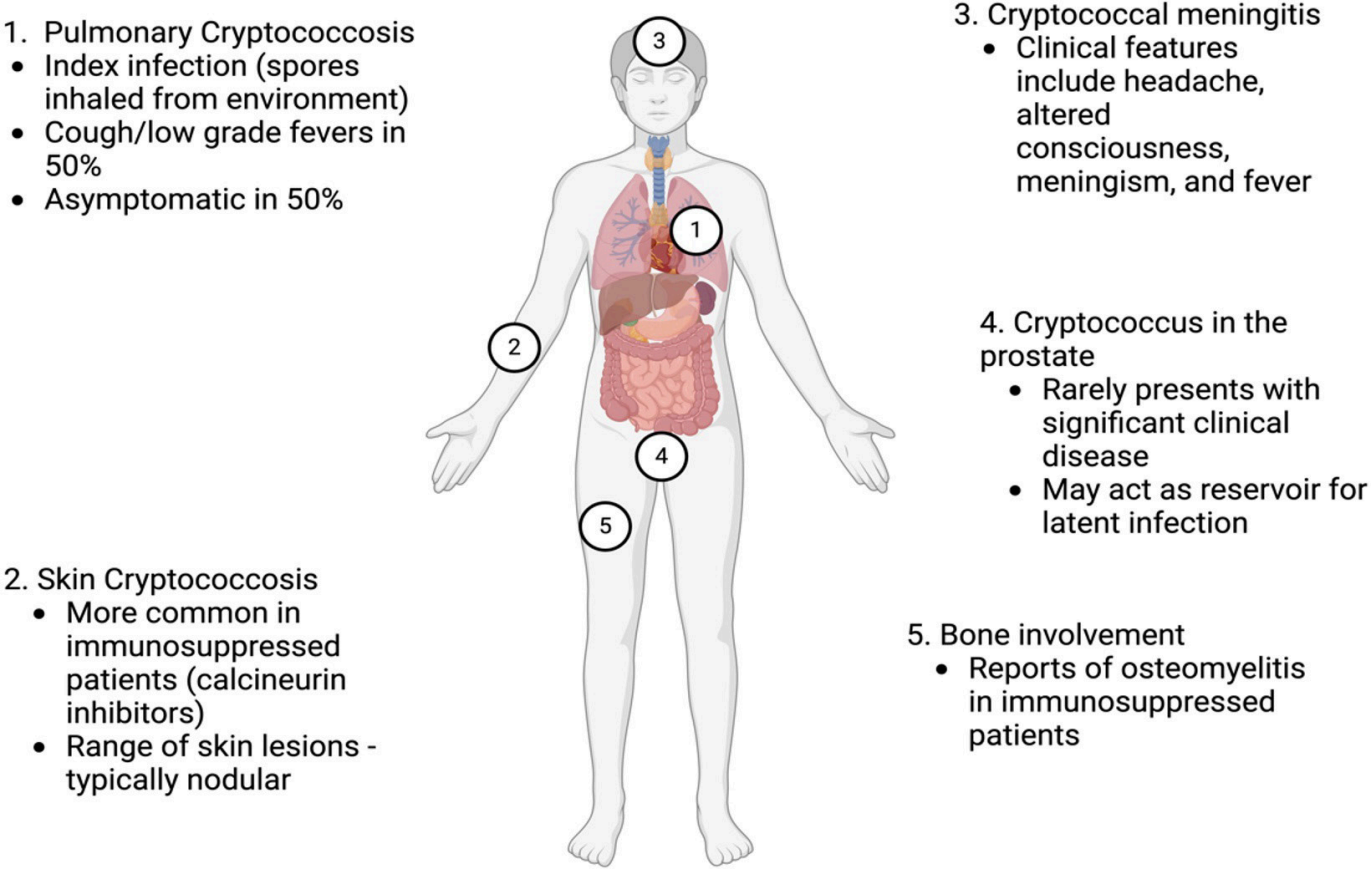
Cryptococcosis as a multisystem disease. Ubiquitous *Cryptococcus* spores in the environment are inhaled and exposed to alveolar macrophages. They can cause primary lung infection, with subsequent dissemination to other sites, including skin, bone, prostate and brain.

*Cryptococcus* is a yeast found in soil, decaying wood and avian excreta. Cryptococcal biology is reviewed elsewhere^6^ but, in brief, ubiquitous cryptococcal spores are inhaled and then exposed to alveolar macrophages. This may lead to primary pulmonary cryptococcosis, which typically manifests as a self-limiting lower respiratory tract infection with low-grade fevers and cough (figure 2). However, as in tuberculosis (TB), probably up to 50% of primary infections are asymptomatic.^7^ Although CM pathophysiology remains incompletely understood, there is clearly a marked heterogeneity in progression to CM following primary infection. This largely reflects host risk factors. Some patients—particularly people living with HIV—may progress rapidly to CM following primary infection, while others have a prolonged incubation period or never develop clinical disease. The pathogen may reside within phagocytes for years with estimated incubation periods of 110 months for *Cryptococcus neoformans* and 24 months for *Cryptococcus gatti*.^8^ Most cases probably arise from immunosuppression triggering conversion from latent to active secondary infection.^6^ This is similar to other opportunistic infections, including cytomegalovirus and TB. *Cryptococcus* provokes disease through local immunomodulation, evasion of phagocytic clearance and neurotropism to nutrient-rich cerebrospinal fluid (CSF).^6^ There appears to be a ‘Goldilocks phenomenon’ in the immune response, reflecting host and pathogen factors, whereby CM arises at either end of a spectrum of immune responses. Immunosuppressed patients may mount an inadequate T-lymphocyte response to cryptococcosis within the lungs or skin, allowing systemic dissemination while immunocompetent patients may mount an excessive response to virulent strains.^7^

Globally, around one-third of CM cases occur in HIV-negative (non-HIV CM) patients. This is a heterogeneous group, beset by delays in diagnosis and high mortality, even in high-income countries with widespread access to diagnostics and first-line treatments. Various forms of immunocompromise increase the risk of CM in HIV-negative adults. Nonetheless, a proportion of non-HIV CM patients are immunocompetent, and clinicians should, therefore, consider CM in all cases of lymphocytic meningitis. In large cohort studies of non-HIV CM, there were no underlying predisposing factors in 30% of US patients and 67% of Chinese patients.^9 10^ However, subsequent work hints at subtle underlying immunodeficiencies in this ‘immunocompetent’ group, such as elevated GM-CSF autoantibody levels,^11^ idiopathic CD4-cytopenia, sporadic monocytopenia and monogenic disorders of T and NK cell signalling and antibody function.^12^

**Figure 3:**
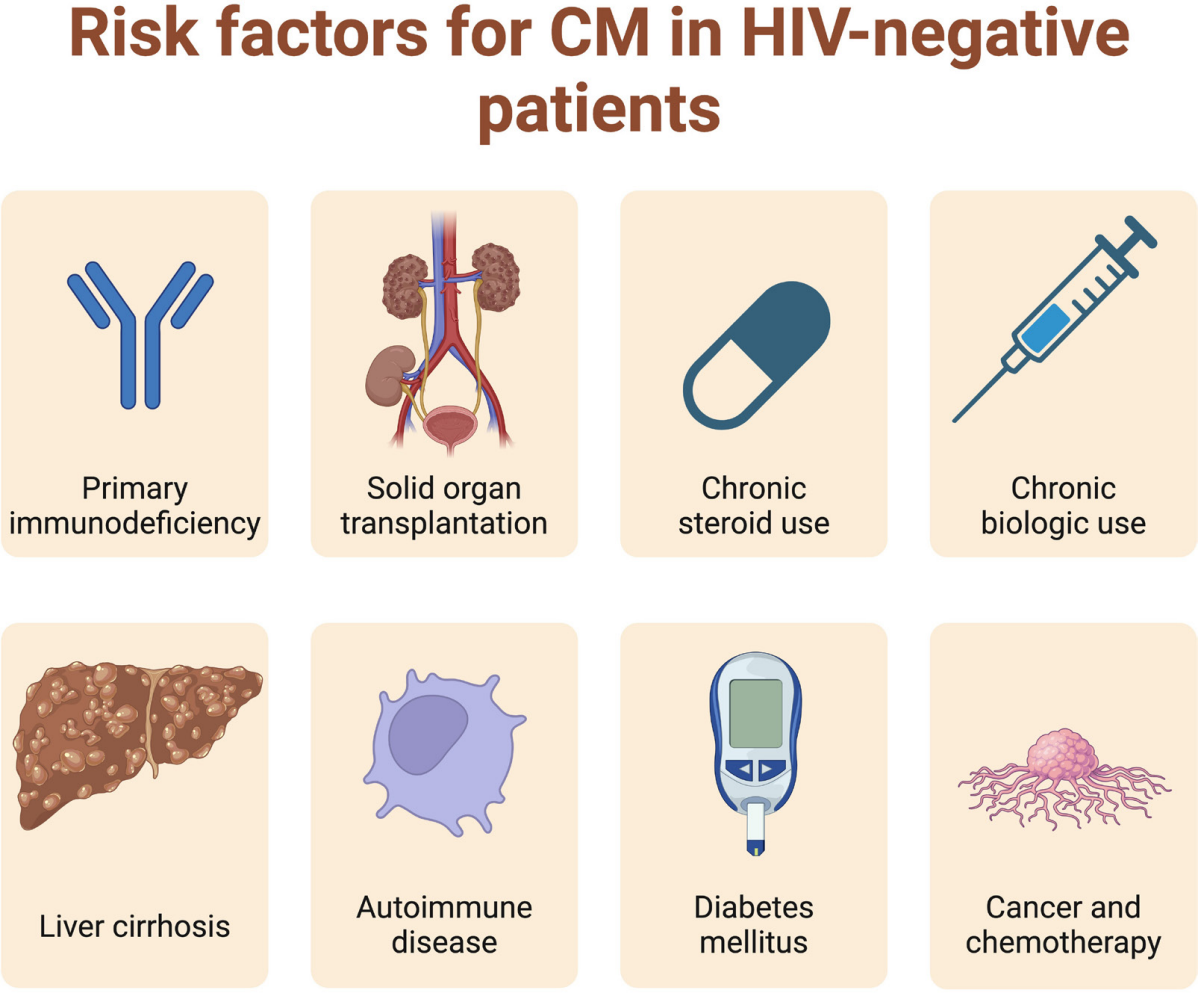
Risk factors for cryptococcal meningitis (CM). Globally, most cases of CM are associated with HIV. While CM can arise in immunocompetent patients, it is unsurprising that the major risk factors for CM in HIV-negative patients are various forms of immunosuppression.

Figure 3 outlines risk factors for non-HIV CM. These include chronic corticosteroid and biologic drug use; organ transplantation; malignancy and chemotherapy; diabetes mellitus and liver cirrhosis. Our clinical experience is that age at diagnosis is often higher in non-HIV CM than in HIV-CM, and some observational studies have identified advancing age as a risk factor for CM and also for mortality from CM.^13 14^ However, this may be confounded by the higher prevalence of comorbidities and prescription of immunosuppressant drugs in older adults. We need further work to determine whether age-related immunosenescence contributes significantly to CM risk and adverse outcomes in older adults.

Corticosteroids potentiate fungal growth, so before starting high-dose corticosteroid therapy, it is important specifically to exclude cryptococcosis and other systemic fungal infections, if these are in the differential diagnosis. Solid organ transplant recipients account for 15%–20% of non-HIV CM cases;^10^ cryptococcosis is the third most common fungal infection in this group after invasive candidiasis and aspergillosis.^15^ CM is typically a late complication of transplantation (with a median time to diagnosis of 20 months) and mortality exceeds 50% in patients with central nervous system (CNS) involvement. Nonetheless, solid organ transplant recipients have a low absolute risk of CM, with an annual incidence of approximately 1/500.

CM also occurs in non-HIV, non-transplant (NHNT) patients. It is more common in people with autoimmune and inflammatory conditions, notably systemic lupus erythematosus, rheumatoid arthritis and sarcoidosis. This probably reflects both disease-related and drug-related immune dysregulation. Anti-TNF agents are relatively over-represented in non-HIV CM cohorts,^16^ and there are case reports of cryptococcosis in patients receiving other biologics, such as rituximab^17^ and tocilizumab.^18^ Despite only limited long-term follow-up data, clinical experience suggests that CM is a rare complication among patients on biologics, given their widespread use in patients with inflammatory gastrointestinal, dermatological and rheumatological conditions. Calcineurin inhibitors, such as tacrolimus, do not appear to increase cryptococcosis incidence but may alter disease manifestations, with lower rates of CM and higher rates of skin, soft tissue and bony involvement.^19^ Many immunosuppressed patients with non-HIV CM have a compelling need for ongoing immunosuppression, but expert consensus is that immunosuppressants should be gradually withdrawn where possible. While there is insufficient high-quality evidence for specific weaning plans, we would advise against abrupt withdrawal (notably of calcineurin inhibitors, mycophenolate and prednisolone^20^) to mitigate the risk of precipitating paradoxical inflammatory treatment reactions and suggest first reducing corticosteroid doses in patients taking multiple agents.

Patients with cancer account for around 25% of non-HIV CM cohorts. Cryptococcosis manifestations vary with cancer type; pulmonary and skin involvement is more common in solid organ tumours and meningitis in haematological malignancy.^21^ A large multicentre registry of Japanese patients with haematological malignancy showed that cryptococcosis accounted for 2.8% of invasive fungal infections, with a low annual incidence of 1/1000.^22^ Although rare even in this population, CM incidence in patients with haematological malignancy is projected to increase as more patients access T-cell depleting chemotherapies. Lymphomas and leukaemias account for most haematological malignancies in CM case series, and CM is rare in allogeneic and autologous stem cell transplant recipients.^23^

### Clinical phenotype

Clinical manifestations of CM vary with *Cryptococcus* species and host factors. Recently published consensus global guidelines split patients into three groups—HIV-CM, solid organ transplant recipients and non-HIV, non-transplant patients.^24^ Clinical features are broadly similar across all groups (subacute or chronic meningitis) but the relative frequency of symptoms and signs varies significantly. Headache is the primary feature in HIV-CM, with a median duration of 3–4 weeks. About half of the cases also have a subjective fever, nausea and vomiting. Meningism is common (75%) but only 25% of patients have objective evidence of fever >38°C. Drowsiness and confusion, which often reflect raised intracranial pressure (ICP), are late signs, emerging 2 weeks after headache onset. In contrast, in HIV-negative cases the median symptom duration before clinical presentation is 6–12 weeks. While there is substantial heterogeneity in immune status and clinical presentation within this group, respiratory symptoms appear to be more common than in people living with HIV.^25^ Headache is less common (reported by around half of patients), but fever, focal neurological deficits and skin lesions are more prevalent in this group than in people living with HIV.^26^ A retrospective cohort study involving 709 North American and Australian patients identified few species-specific differences in clinical features between patients with *C. neoformans* and *C. gattii* CNS disease.^27^ Headache was more common in *C. gattii* cases and altered mental status and nausea more common in *C. neoformans* cases. However, pulmonary lesions are significantly more common in *C. gattii*.

### Diagnosis

**Figure 4:**
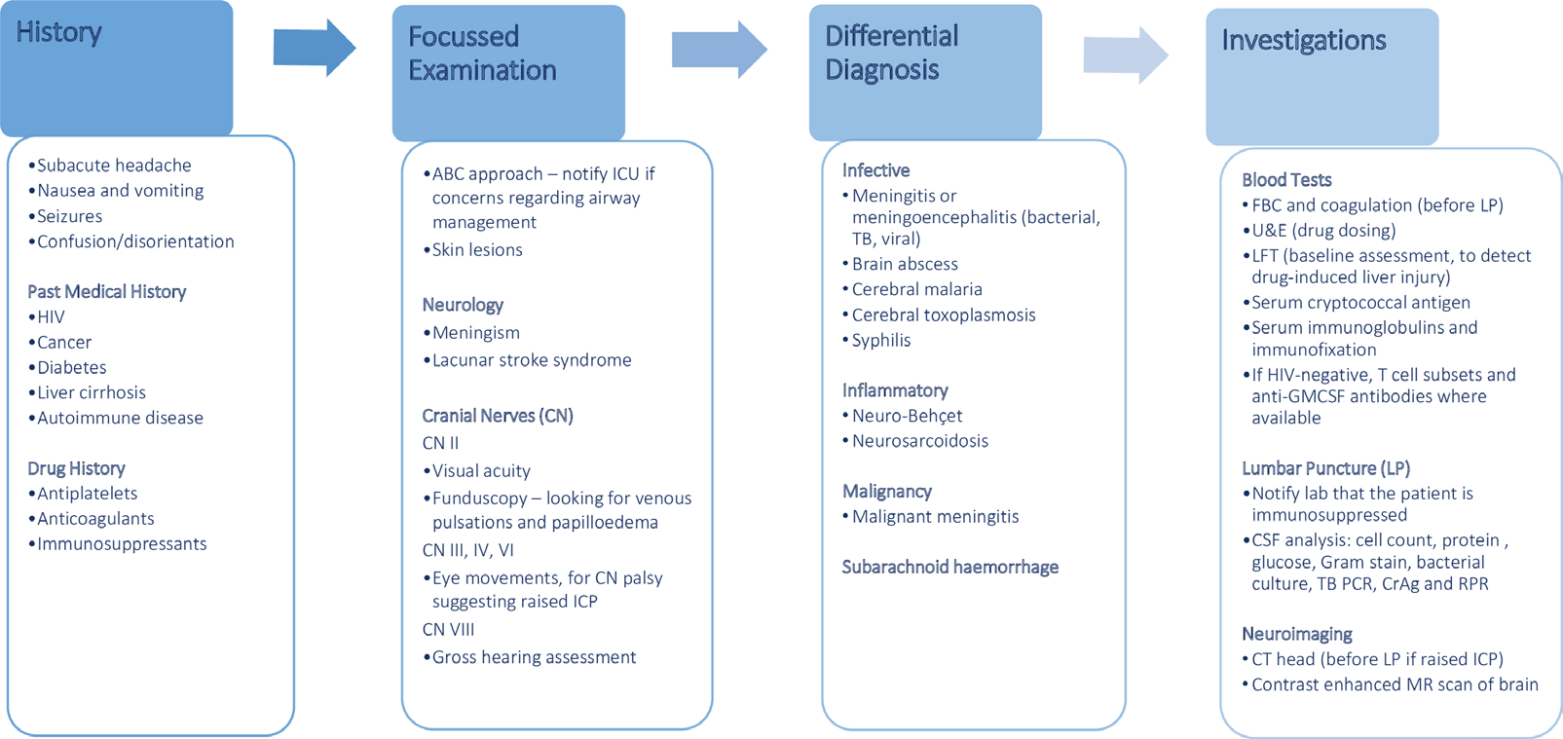
Focused clinical assessment of suspected cryptococcal meningitis. ABC, airway, breathing, circulation; DILI, drug-induced liver injury; FBC, full blood count; GFMCSF, granulocyte-macrophage colony-stimulating factor; LFT, liver function tests; RPR, rapid plasma reagin; TB, tuberculosis; U&E, urea and electrolytes.

**Figure 5:**
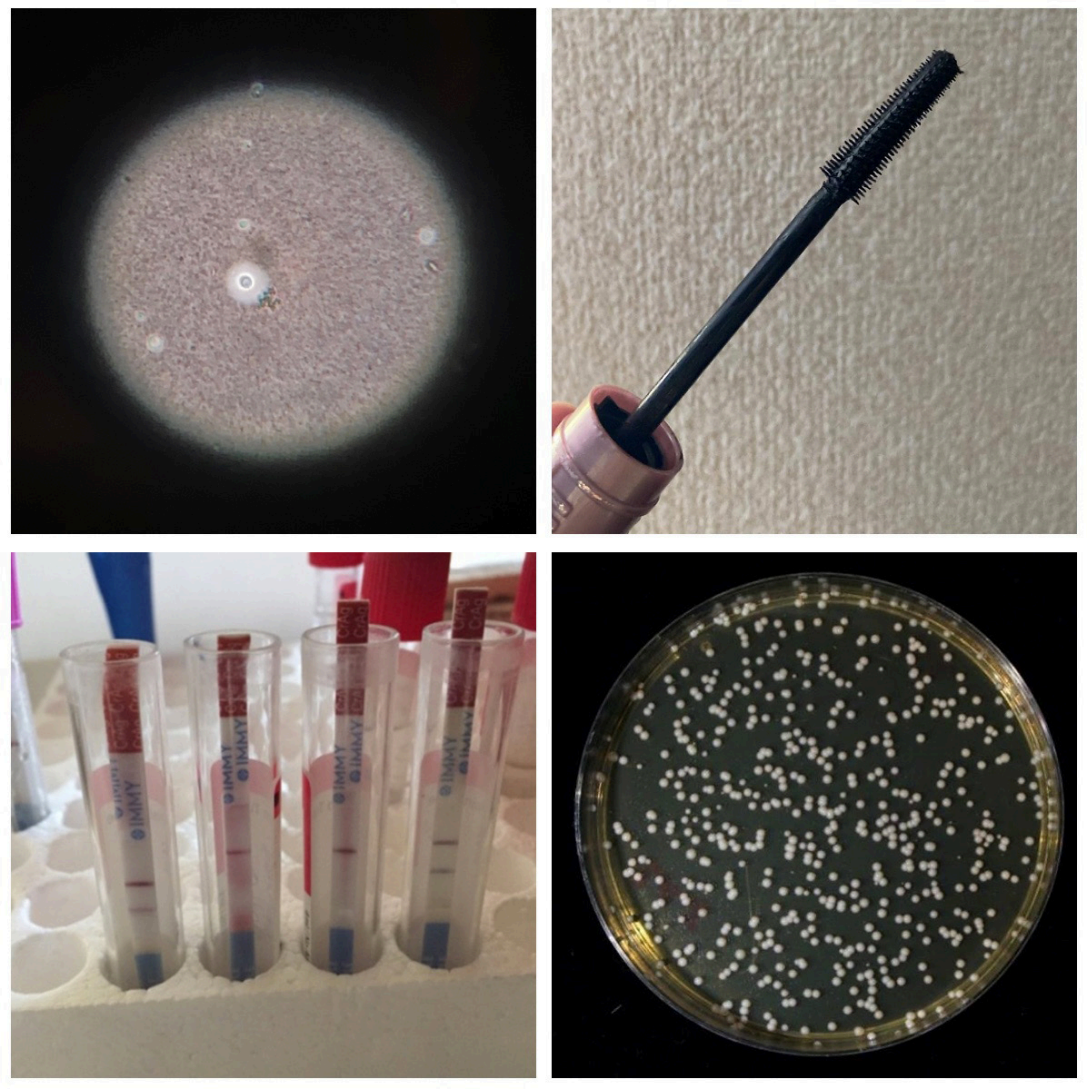
Diagnostic tests in cryptococcal meningitis. Clockwise from top left: (1) light microscopy with India ink staining, demonstrating encapsulated yeast cells within cerebrospinal fluid (CSF). With a sensitivity of 86% in CM, India ink microscopy misses around 1/7 true cases and has now been superseded by cryptococcal antigen testing. (2) However, the supply of cryptococcal antigen lateral flow assays, and even India ink, to rural, low-resource settings, may be interrupted. Mascara is a cheap, widely available, alternative negative stain. (3) *Cryptococcus neoformans* colonies on a yeast-extract peptic dextrose (YPD) agar plate. Fungal culture is essential in cases of clinical relapse, to differentiate between true microbiological relapse and inflammatory treatment reactions. (4) Cryptococcal antigen lateral flow assays. These assays offer a rapid, qualitative (positive/negative) result and can be performed at the bedside on blood and CSF samples. They have excellent sensitivity and specificity in symptomatic patients and generally constitute the diagnostic test of choice in suspected CM.

Figure 4 outlines a plan for approaching patients with suspected CM, encompassing clinical features, differential diagnosis and basic investigations. Box 1 presents practical diagnostic tips, and figure 5 shows images of important diagnostic tests. CM diagnostics have been transformed in recent years, but it is still useful to know the characteristic findings of CSF analysis. These include elevated opening pressure in the left lateral position (sitting lumbar puncture (LP) pressures are unreliable), lymphocytic pleocytosis, high CSF protein and low CSF glucose. In HIV-CM, the CSF white cell count may be lower than expected, or even normal, so it is important to perform specialised tests as outlined in table 1. The recommendations made in table 1 are adapted from the British Society for Medical Mycology 2015 best practice guidelines.^28^

Box 1 **Diagnostic tips**

- Think about cryptococcal meningitis (CM) if your patient’s cerebrospinal fluid (CSF) shows abnormal white cell count (especially lymphocytosis), glucose or protein concentrations.
- In a UK setting, for the correct CSF tests to be performed, it is important to specify that a patient is immunocompromised on the test request, or notify the laboratory.
- India ink microscopy is no longer recommended unless cryptococcal antigen (CrAg) lateral flow assays are unavailable.
- Serum and CSF CrAg are qualitative lateral flow tests with high sensitivity and specificity—in symptomatic patients, negative CSF CrAg effectively rules out CM.
- Once positive, CrAg remains so for many months—it cannot be used to monitor treatment response or to diagnose early CM relapse.
- Fungal culture can be helpful for diagnosis, but treatment is not usually contingent on antifungal sensitivities; it is, therefore, most useful in CM recurrence and relapse.
- Where possible, a contrast-enhanced MRI brain should be performed within 48 hours in immunocompromised patients with suspected or proven meningitis and/or new neurological features such as seizures, persistent headache and altered mental state.

**Table 1:** Diagnostic tests

Diagnostic test	Advantages	Disadvantages and pitfalls	Recommendations
India ink microscopy	Provides rapid result within 10 minRelatively cheap *cf*. culture methods	Operator-dependent—requires expertise in microscopyLower sensitivity and specificity for CM than CrAg—particularly in patients with lower fungal load (early in disease course/on ART)Does not provide information on species or antifungal sensitivities	Do not perform unless CrAg unavailable on site
Cryptococcal antigen (CrAg) lateral flow assay	Very high sensitivity and specificity in symptomatic patients—confers high negative predictive valueProvides rapid result within 10 minCan be performed by staff with minimal clinical and laboratory training—making it cheap and scalableCan be performed on serum samples in patients in whom CSF sample difficult to acquire	Remains positive for months—not useful for monitoring treatment response or diagnosing relapseTitres connoting ‘positivity’ vary between manufacturersDoes not provide information on species or antifungal sensitivities	Perform CrAg in all patients with CSF high in lymphocytes and protein and/or low in glucose without an adequate explanationIn immunosuppressed patient with contraindication to LP, perform serum CrAgDo not use CrAg to monitor treatment response or diagnose relapse
Fungal culture	Identifies viable cells capable of reproduction, signalling active infection when specimen obtained from sterile siteHigh sensitivity and specificity in symptomatic patientsProvides detailed information on *Cryptococcus* species and drug sensitivities	Need for highly-trained staff—expensive and challenging in low-resource settingsUnless laboratory is aware that patient is immunocompromised, fungal culture is not carried out routinely—need to notifyCalls for energy-intensive and expensive incubation equipment, which may not be available in low-income settingsRisk of sterile culture if sample obtained some time after treatment started—false negative result	Where available, fungal culture should be performed for 21 days for all immunocompromised patients and people with sarcoidosis or cancer, with abnormal leucocyte count, protein and/or glucose in CSFCSF fungal culture is the investigation of choice in suspected CM relapseUnless dealing with disease recurrence, antifungal sensitivities have little bearing on treatment
Galactomannan	Readily available fungal biomarker—detectable in both serum and CSF	Low sensitivity for CM—most useful in suspected invasive aspergillosisModerate wait for results—in-house testing of serum samples usually gives result in 24–48 hours	Do not use in assessment of suspected CM
Beta D-glucan	Readily available fungal biomarker—detectable in both serum and CSF	Pan-fungal marker—not specific to CMModerate wait for results—in-house testing or serum and CSF samples usually gives results in 24–48 hours	Do not use in assessment of suspected CMConsider CM as possible cause of raised serum BDG in patients with compatible syndrome

ARTantiretroviral therapyBDGbeta D-glucanCMcryptococcal meningitisCSFcerebrospinal fluidLPlumbar puncture

India ink microscopy, a negative staining technique which was once the mainstay of CM diagnostics, has been superseded by cryptococcal antigen (CrAg) lateral flow assays. However, maintaining reliable supply chains for CrAg tests, and even basic laboratory reagents such as India ink, is challening in low-resource, high-prevalence settings, especially in the context of conflict and catastrophe. If CrAg tests are unavailable, and India ink supplies run low, mascara is a widely available, cheap alternative.^29^

CrAg is a qualitative test that provides a rapid, binary positive or negative result, with high positive and negative predictive value in symptomatic people living with HIV (99% sensitive and 95% specific for serum and 99% sensitive and 99% specific for CSF samples).^30^ CrAg is not operator-dependent and can be performed by staff without microscopy training. Importantly, in susceptible hosts, serum CrAg may be positive for several weeks before symptom onset.^31^ Cryptococcal antigenaemia, therefore, represents a targetable ‘predisease’ state and countries including Uganda, South Africa and Zimbabwe have implemented routine, systematic, asymptomatic serum CrAg testing in people living with HIV whose CD4 count is <200 cells/µL.^32^ At present, a positive serum CrAg on asymptomatic screening mandates an LP, as a positive CSF CrAg connotes CM and alters clinical management. In patients with a CNS syndrome compatible with CM, but a compelling contraindication to LP, serum CrAg positivity alone should prompt antifungal treatment.^33^ This may evolve as next-generation semiquantitative CrAg assays enter the market, helping clinicians to risk-stratify patients, with those with low serum CrAg titres potentially avoiding LP.^30 34^

Multiplex PCR-based rapid diagnostic tests are commonly used to determine the cause of suspected CNS infection in high-income settings. The BioFire meningitis/encephalitis panel contains a single conserved target for *C. neoformans* and *C. gattii* and targets for 13 other pathogens. This test remains out of reach in most low-income and middle-income countries, but as costs fall, its use will become more widespread within the African meningitis belt, where it will help clinicians to distinguish rapidly between CM, bacterial meningitis and cerebral malaria. A large Ugandan study compared the diagnostic performance of BioFire meningitis/encephalitis against CrAg and fungal culture in 328 adults and 42 children with CM.^35^ Although highly specific (98%), the sensitivity of the BioFire meningitis/encephalitis panel for *Cryptococcus* was just 82%, with CrAg as the reference standard. A positive test is useful and should prompt treatment, but a negative test does not rule out CM, and such patients require additional CrAg testing to rule out CM.

CSF fungal culture is the gold standard for CM diagnosis, signalling viable yeast cells capable of reproduction. However, culture requires skilled staff and expensive equipment, which precludes its widespread use in low-income settings. CSF fungal culture is necessary for suspected relapse as CrAg can remain positive for weeks or months, and microbiocidal sensitivities are needed to rule out treatment failure due to antifungal resistance. In the UK, CSF is routinely cultured on bacterial media, rather than dedicated fungal cultures; a clinician can specifically request fungal cultures if needed. As such, cryptococcal growth on standard CSF cultures is often scant, but even ‘scant *Cryptococcus* growth’ is alarming given it has occurred on suboptimal culture plates from a sample obtained from a usually sterile site. Although *Cryptococcus* is ubiquitous, contamination is rare and thus scant growth on bacterial culture medium effectively confirms the diagnosis and should prompt urgent treatment. We do not recommend routine testing of serum and CSF samples for additional fungal markers such as galactomannan and beta D-glucan, which are limited by poor sensitivity and specificity.^36 37^

### Neuroradiology

Neuroimaging is a useful adjunct to CM diagnosis and is important for identifying complications such as hydrocephalus, cryptococcomas and stroke. It is particularly important in HIV-CM as people living with HIV may have concurrent opportunistic CNS infections. Plain CT imaging may be necessary in the hyperacute period (eg, to exclude important differential diagnoses for coma) but cannot exclude CM. Neuroimaging abnormalities in CM are often subtle and the preferred modality is contrast-enhanced MRI. This is reflected in British Society for Medical Mycology guidelines, which advocate for urgent MR scan of the brain within 48 hours for immunocompromised patients with new neurological features such as new persistent headache, altered mental state, or seizures, and those with possible or proven meningitis. If this is unavailable, contrast-enhanced CT head is the next best option.^28^

**Figure 6:**
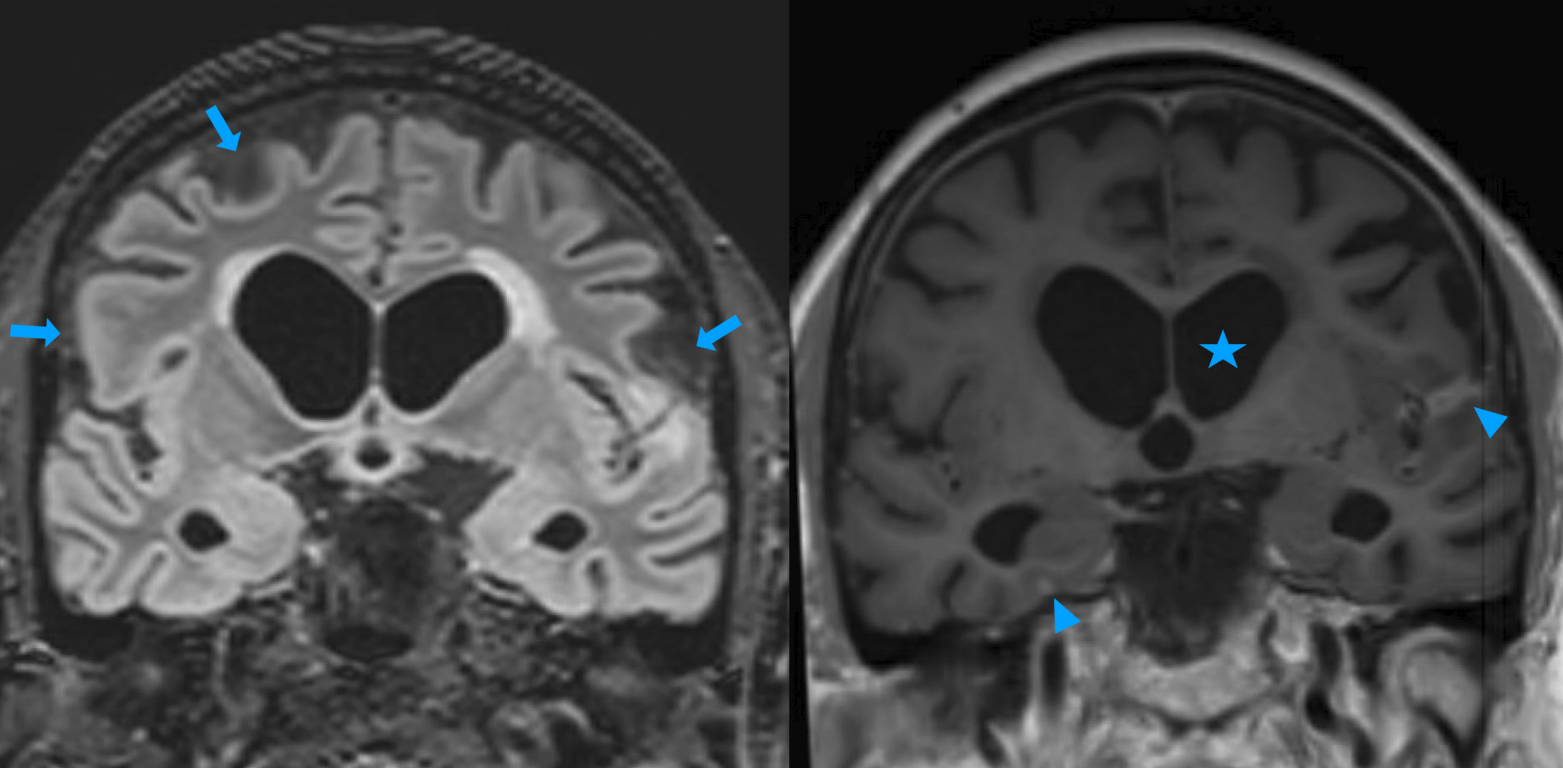
MRI sequences from an immunosuppressed adult with haematological malignancy. Coronal FLAIR (left) and post gadolinium T1w (right) images show hydrocephalus (star), increased signal in CSF spaces over the cerebral convexities ‘dirty CSF‘ (arrows) and subtle meningeal enhancement (arrowheads). Reprinted from The BMJ Case Rep (Internet). 2023 December 7;16(12):e257720. Coughlan *et al*, ‘Dirty CSF’: an MRI feature of CNS fungal infection. Copyright (2023), with permission from BMJ Publishing Group.^38^ CNS, central nervous system; CSF, cerebrospinal fluid; FLAIR, fluid-attenuated inversion recovery.

Characteristic neuroimaging findings include dilated Virchow-Robin spaces, the ‘dirty CSF sign’ (figure 6),^38^ hydrocephalus, cryptococcomas and hazy brain base sign. Many of these findings are non-specific, but the presence of basal meningeal enhanacement is significant as it is associated with the future development of cerebral infarcts.^39^ As outlined below, hydrocephalus indicates a poor prognosis and should prompt urgent discussion with a neurosurgeon.

### Management

The central tenets of CM management are aggressive fungicidal treatment at diagnosis followed by long-term fungistatic treatment to achieve clearance and suppression; and proactive management of raised ICP, which develops in up to 75% of patients. Online supplemental material 1 discusses medical management of CM in depth. Table 2 outlines the side effects of antifungal agents used in CM and gives a treatment algorithm based on consensus global guidelines.^24^ Box 2 lays out important principles of CM management.

**Table 2:** Antifungal treatment guidelines

Drug		Amphotericin B	Flucytosine	Fluconazole
Mechanism		Fungicidal polyene that creates pores in fungal cell membranes	Fungistatic RNA analogue that disrupts yeast replication	Fungistatic azole that inhibits fungal cell wall synthesis
Phase		Induction		Consolidation and maintenance
Cryptococcal antigenaemia		Generally omit	Generally omit	6–12 months of oral fluconazole monotherapy.Pregnancy—seek expert advice
Dose and duration	HIV	Low-income setting—single-dose intravenous liposomal amphotericin B (10 mg/kg)*High-income setting—2 weeks liposomal amphotericin B (3–4 mg/kg/day)	100 mg/kg/day for 14 days split into four divided doses.	Low-income setting, induction phase 1200 mg once daily for 1 weekConsolidation therapy—all settings400–800 mg once daily for 8 weeksMaintenance therapy—all settings200mg once daily until viral suppression achieved and CD4 >200 cells/mm^3^)
	SOT	2 weeks low-dose intravenous liposomal amphotericin B (3–4 mg/kg/day)		Consolidation therapy—all settings400–800 mg once daily for 8 weeksMaintenance therapy—all settings200 mg once daily for 6–12 months
	NHNT			
	Pregnancy	Intravenous liposomal amphotericin B (1 mg/kg/day) for the first 2 weeks; then seek expert advice	Consider on case-by-case basis but generally omit	Relatively contraindicated.Safe in breastfeeding
Adverse effects		Infusion reaction—chills and rigors (common) Nephrotoxicity and hypokalaemia (common) Hepatotoxicity (common)	Neutropenia (4%) and myelosuppression	Dry skin, alopecia, diarrhoeaTransient liver function test (ALT) derangement)
How to detect and mitigate adverse effects		Use liposomal formulationSlow infusion rate (3–6 hours)Prehydration with intravenous fluids. Baseline U&E, repeated twice weekly	Baseline full blood count and urea and electrolytes, then twice weekly Reduce dose in renal impairment Check flucytosine levels weekly	Reduce dose if adverse effects (particularly in women)If intolerant, can switch to itraconazole

* This is a recent change in the medical management of cryptococcal meningitis recommended by WHO and a global consensus guideline, based on the AMBITION-cm trial. Please refer to online supplemental material 1 for a detailed discussion.

ALTalanine transaminaseNHNTnon-HIV, non-transplantSOTsolid organ transplantU&Eurea and electrolytes

Box 2 **Principles of cryptococcal meningitis (CM) treatment**

- All patients require prolonged courses of antifungal treatment, usually lasting 6–12 months—split into induction, consolidation and maintenance phases.
- Aggressive, combination fungicidal therapy during the induction phase is associated with reduced risk of acquired antimicrobial resistance and reduced mortality.
- Once the disease is controlled, the patient can be transitioned to fungistatic treatments (fluconazole), which have good oral bioavailability and central nervous system penetration.
- For HIV-CM in low-resource settings, induction therapy with single-dose intravenous liposomal amphotericin B and oral flucysotine 25 mg/kg four times per day for 1 week, followed by high dose oral fluconazole 1200 mg once daily for a further week, is non-inferior to a 1-week liposomal amphotericin B -based induction period, reducing care costs and side effect burden.
- There is no established role for adjuncts; corticosteroids are reserved for immune reconstitution inflammatory syndrome (IRIS), postinfectious inflammatory response syndrome and cryptococcomas.
- In non-HIV CM cases with a compelling need for ongoing immunosuppression, avoid abrupt medication withdrawal to mitigate the risk of paradoxical treatment reactions and wean corticosteroids first.
- In CM patients with a new diagnosis of HIV, delay antiretroviral therapy for 4–6 weeks after starting antifungals to mitigate the risk of IRIS.
- The key complication of CM is raised intracranial pressure, which may not be present at time of diagnosis—all patients should undergo early repeat lumbar puncture and may require serial therapeutic taps or cerebrospinal fluid diversion.
- Where possible, all CM patients should be discussed in a multidisciplinary team meeting involving neurologists, infectious disease physicians, neurosurgeons, neuroradiologists, therapists and pharmacists.

### Neurosurgical involvement/raised ICP

**Figure 7:**
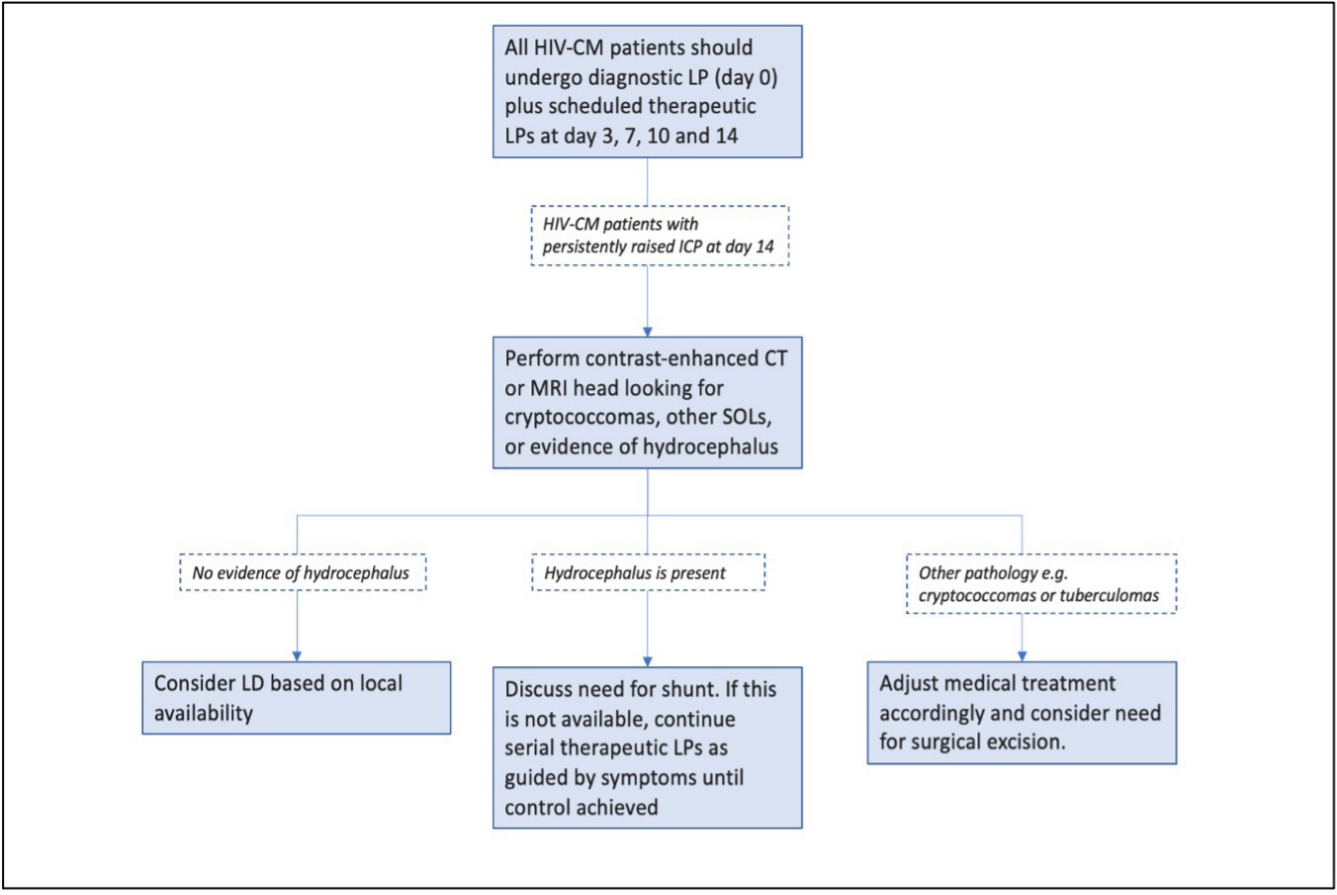
Raised ICP management in cryptococcal meningitis. CM, cryptococcal meningitis; ICP, intracranial pressure; LD, lumbar drain; LP, lumbar puncture; SOL, space occupying lesion.

Raised ICP is a sign of disease severity, associated with increased morbidity and mortality. This appears to be driven by distinct pathophysiological processes in people living with HIV and non-HIV CM. Aggressive management of raised ICP improves patient outcomes.^12^ Box 3 defines key terms pertaining to CSF dynamics with important therapeutic implications. Figure 7 outlines our suggested approach to raised ICP management in CM.

Box 3 **Working definitions for key neurosurgical terms**

- Raised intracranial pressure (ICP)—a cerebrospinal fluid (CSF) opening pressure exceeding 20 cm H_2_O at time of lumbar puncture in the left lateral position.
- Ventriculomegaly—an increase in ventricular dimensions on neuroimaging/pathological analysis, which frequently occurs as a compensatory response to raised ICP.
- Hydrocephalus—a disturbance in CSF dynamics that commonly manifests as a dyad of clinical features of raised ICP and radiological evidence of ventriculomegaly.

Treatment options for raised ICP and hydrocephalus in CM include serial therapeutic LPs; temporary external CSF diversion through lumbar drain or external ventricular drain insertion; or definitive CSF diversion with tunnelled ventricular shunts. While there are various forms of shunt surgery, ventriculoperitoneal shunt insertion is the most widely practised in CM. In skilled hands, this is a routine procedure, but it requires designated theatre space and neurosurgical expertise. Long-term cumulative risks of shunt obstruction also mandate long-term neurosurgical follow-up. In low-income settings, lumbar drain insertion can be performed at the bedside by surgical associates without postgraduate neurosurgical training. Lumbar drain insertion allows short-term, safe, continuous drainage of larger volumes of CSF than LP (up to 200 mL CSF/day vs 30 mL/day with LP) and lumbar drain-related infection is uncommon, even in low-resource settings.

The pathophysiology of raised ICP and hydrocephalus in CM is not completely understood. In HIV-CM, there is a suppressed inflammatory response and high fungal burden, which seems to be necessary, but not sufficient, for the development of raised ICP.^40^ Autopsy studies of CM in people living with HIV have identified fungal and cellular debris at the arachnoid granulations, which impedes CSF reabsorption.^41^ Interestingly, these patients tend to develop raised ICP in the absence of hydrocephalus. Approximately 50% of HIV-related cases develop raised CSF opening pressures during their illness (defined as a pressure exceeding 25 cm H_2_O), with 25% having severely raised pressures over 35 cm H_2_O.^42^ While there is often raised ICP at baseline LP, it can occur several weeks into antifungal treatment, underlining the need for close follow-up. Fungal burden and raised ICP improve rapidly with fungicidal therapy in people living with HIV. Despite some negative studies, there is now convincing evidence that therapeutic LPs reduce mortality in HIV-CM.^43 44^ This is reflected in WHO guidelines, which advocate for early repeat LP in all patients (to ensure fungal clearance and normal opening pressure); and serial therapeutic LPs in patients with raised baseline OP>25 cm H_2_O and in patients with persistent signs or symptoms of raised ICP after starting treatment.^45^ However, a recent Ugandan study^43^ involving 533 adults with HIV-CM highlighted significantly raised 30-day mortality in patients who did not receive at least one therapeutic LP, irrespective of opening pressure at diagnostic LP. The authors suggest that we should move away from using baseline opening pressure and symptoms as indications for therapeutic LP and instead perform scheduled LPs on day 3, 7, 10 and 14 in all subgroups of CM. If patients are discharged within 7 days of admission, we suggest maintaining this schedule of therapeutic taps in an outpatient setting, where feasible. Patients with raised ICP should ideally remain in the hospital until the opening pressure has normalised, preferably until after day 14. If such patients wish to self-discharge due to the high costs of inpatient care, we would suggest regular outpatient taps every 3–4 days.

Most CM cases in people living with HIV improve with antifungal treatment±(serial) therapeutic LPs, but a proportion require CSF diversion. Historically, lumbar drain has been preferred in this subgroup as most patients require only short-term drainage for 5–10 days. There are few reports of obstructive hydrocephalus in HIV-CM, and permanent CSF diversion is rarely required. In contrast, patients with non-HIV CM have higher rates of hydrocephalus and higher odds of requiring neurosurgical intervention. The mechanisms underlying hydrocephalus in this group are poorly understood, and further work is needed to elucidate contributory host/pathogen interactions. However, in a significant number of non-HIV CM patients, hydrocephalus may arise as a result of choroid plexitis, with associated narrowing of the fourth ventricle.^12^

In middle-income and high-income countries, shunting is performed in 15%–20% of patients with CM. Retrospective cohort studies in Thailand^46^ and the USA^47^ have identified hydrocephalus, raised baseline CSF opening pressure and non-HIV status as factors associated with higher odds of ventriculoperitoneal shunt insertion. In the US cohort, immunocompetent patients had an OR of 6.3 for ventriculoperitoneal shunt insertion. Interestingly, this study found increased odds of ventriculoperitoneal shunt insertion in female patients, which is supported by similar findings in a study of 50 US adults with CM (98% of whom were living with HIV).^48^ A small, older study involving 10 adults with CM suggests that ventriculoperitoneal shunt insertion is safe and effective, with no seeding of cryptococcal infection to the peritoneum or bloodstream, or persistent biofilm formation provided fungicidal therapy had already been started.^49^

Where available, we suggest using shunts with valves that can be manually adjusted to optimise CSF drainage in the face of changing drainage rate requirements. Certain adjustable valves allow surgeons to reduce CSF drainage gradually and thereby ascertain whether the patient remains shunt-dependent or can be gradually weaned off the shunt. The rationale for this approach is that CSF dynamics might recover with medical treatment, with no further need for artificial diversion, allowing safe shunt removal. This is typically performed once patients have transitioned to maintenance therapy or completed treatment.

Many studies advocate for ‘timely’ neurosurgical intervention, but none define this clearly. Careful patient selection is likely to be key. Where possible, all patients should undergo early neuroimaging and manometers should be used to measure opening pressure at baseline and subsequent LPs. We suggest routine discussion of all CM cases with neurosurgical colleagues to identify candidates for early intervention. As raised ICP rapidly improves with medical therapy in most HIV-CM patients—tempering the benefits of shunt surgery—we would generally advocate for performing scheduled therapeutic LPs and avoiding neurosurgical intervention in this group. In comparison, immunocompetent patients with raised ICP>30 cmH_2_O and evidence of hydrocephalus are highly likely to require long-term CSF diversion and we would favour intervention in this group shortly after their initial diagnostic LP. In low-income settings, people living with HIV requiring repeated taps beyond 10 days of treatment initiation should be prioritised for neuroimaging (to identify ventriculomegaly and exclude concurrent pathology such as a cryptococcomas) and considered for lumbar drain or ventriculoperitoneal shunt insertion. Following lumbar drain insertion, we would suggest a maximal diversion of 10–15 mL CSF/hour to avoid overdrainage complications. If lumbar drain or ventriculoperitoneal shunt insertion is unavailable or impractical, further therapeutic LPs should be continued until raised ICP symptoms resolve. There are no high-quality studies that have convincingly identified predictors of poor outcomes after shunt surgery in CM, but our experience suggests that patients with fixed, dilated pupils are unlikely to benefit.

### Complications and prognosis

Table 3 summarises common and important complications of CM. Mortality varies with setting, immune status, and pathogen and host factors. In a 2014 cohort study of 501 adults with HIV-CM in low-income and middle-income countries, the 10-week mortality was 34%.^42^ In contrast, a database study involving 1351 patients diagnosed with CM in the USA between 2000 and 2012 found a 1-year mortality of 11.6%.^50^ Predictors of mortality include altered mental state and age over 50 years. Even in the absence of these features, high baseline CSF fungal load and slow fungal clearance are associated with a worse outcome, emphasising the importance of prompt fungicidal treatment.^42^ A single-centre retrospective cohort study involving 302 patients in a US setting identified cryptococcaemia (OR 5.09) and raised ICP (opening pressure >25 cmH_2_O; OR 2.93) as poor prognostic indicators, and HIV seropositivity (OR 0.46) as a good prognostic indicator.^25^ Interestingly, this was mirrored by a US single-centre retrospective cohort study, which found a higher 90-day mortality in non-HIV, non-transplant patients vs HIV-CM (HR 3.3).^51^ Poorer outcomes in non-HIV CM may reflect delays in care seeking and diagnosis in non-HIV, non-transplant patients.^52 53^

**Table 3:** Complications of cryptococcal meningitis

Complication	Approximate incidence	Considerations
ART-associated Cryptococcosis/unmasking IRIS	HIV-CM 1%	Fulminant CM presentation days after initiation of ART with evidence of cryptococcus in CSF. Treat as HIV-CM (see Table)
Paradoxical IRIS	HIV-CM 10%–50%	Occurs 1–12 months after ART initiation. Clinically, resembles CM relapse, with sterile CSF. Expert consensus advocates steroid initiation (varied formulations and doses—discuss with local Infectious Diseases team)
PIIRS	Limited data	20–35 days postinfection in immunocompetent patients. Repeat CSF culture shows no fungal growth. Case reports suggest improvement with steroids, with varied formulations and doses—discuss with local ID team
Cryptococcomas	NHNT up to 40%	50% single lesion, 50% multifocal. 73% have perilesional oedema. Steroids and surgical resection on case by case basisIDSA recommend prolonged induction (6 weeks) and 6–18 months fluconazole
Seizures	HIV-CM 25%,Non-HIV CM 10–20%	Associated with raised ICP, predictor of increased mortality
Stroke	10–30%	Ischaemic infarcts predominantly lacunar. Multiterritorial infarct could indicate CM-associated intracranial vasculitis
Neurocognitive impairment	HIV-CM 90% at 1 month40% at 12 months	Data are confounded by advanced HIV, but this is likely to be under-recognised in survivors and contributes significantly to economic impact of CM on survivors and to wider society
Visual Impairment	HIV-CM 40–50%	Severity correlates with ICP>20 cmH_2_O
Hearing impairment	HIV-CM 30%–90%NHNT 30%	Severity correlated with ICP>20 cmH_2_O

ARTantiretroviral therapyCMcryptococcal meningitisCSFcerebrospinal fluidICPintracranial pressureIDSAInfectious Diseases Society of AmericaIRISimmune reconstitution inflammatory syndromeNHNTnon-HIV, non-transplantPIIRSpostinfectious inflammatory response syndrome

Clinical deterioration in patients established on treatment may herald CM relapse or treatment failure; an inflammatory treatment reaction; stroke or concurrent pathology (especially in immunocompromised hosts). In this scenario, reimaging and repeat CSF analysis for fungal culture are essential. Repeat CSF CrAg does not help and the presence of yeasts on microscopy should be ingored if cultures are sterile, as this implies non-viability. In patients with negative fungal cultures but persistent lymphocytic pleocytosis, one should consider a diagnosis of an inflammatory syndrome. In HIV-CM, starting antiretroviral therapy boosts immune function, improving recognition of fungal antigens and prompting a significant inflammatory response (‘paradoxical’ immune reconstitution inflammatory syndrome (IRIS)).^54^ In a high-quality clinical trial, starting antiretroviral therapy 1–2 weeks after CM diagnosis was associated with increased 10-week mortality versus delayed initiation at 5 weeks.^55^ As such, in HIV-CM, one should measure the CD4 count at diagnosis, defer starting antiretroviral therapy until 4–6 weeks of antifungal treatment, and use the CD4 count to define fluconazole duration. Fluconazole can be stopped once viral suppression has been achieved and CD4 count exceeds 200 cells/µL. Inflammatory reactions also occur in HIV-negative subgroups, often in immunocompetent hosts. This is termed postinfectious inflammatory response syndrome, a rare phenomenon that occurs 3–5 weeks after starting treatment. Clinically, it resembles CM relapse but patients demonstrate fungal clearance.^56^ Consensus global guidelines advocate corticosteroid therapy with close monitoring and management of CSF pressure changes.^24^ Steroid-refractory patients may require cyclophosphamide.

Cryptococcomas are single or multifocal space occupying CNS lesions, more common in *C. gattii*. A more prolonged induction period of 4–6 weeks should be considered in these patients. Corticosteroids are indicated if there is perilesional oedema (seen in 75% of cases) and mass effect^57^; they should be slowly tapered over 4–6 weeks to mitigate the risk of IRIS. Where possible, all cases should be discussed in an multidisciplinary team (MDT) meeting involving a surgeon and neuroradiologist, given the wide radiological differential diagnosis for these lesions (including pyogenic brain abscess, tuberculoma, cerebral toxoplasmosis and neurocysticercosis), and their frequent need for surgical intervention. There are case reports of persistent radiological lesions in patients who have achieved microbiological and clinical cure.^58^

Seizures at first presentation are associated with raised ICP and are an independent poor prognostic indicator. There are few high-quality longitudinal studies of CM survivorship, but clinical experience, and limited follow-up data suggest that while acute seizures are common, post-CM epilepsy is rare.^59^ Ischaemic strokes commonly affect deep lacunar territories at first presentation, but may also arise in multiple territories due to para-infectious intracranial vasculitis.^60^ There are few data on long-term neurodisability after CM, but prevalence is thought to range from 20–70%, with higher odds of neurodisability in those who received inadequate antifungal treatment and ICP management.^52 61^ Reported disability includes neurocognitive impairment, permanent hearing loss, residual headache, vertigo and motor deficits.^62^ Many survivors are of working age, and require significant care from relatives, which underlines the huge indirect economic burden of CM worldwide. Disability typically peaks at 1 month after diagnosis, and improves gradually over the next 12 months. Where possible, all patients should be reviewed by an occupational therapist during their recovery phase to optimize neurorehabilitation, and remain under close follow-up with Infectious Diseases and Neurology, using detailed psychometric testing, and vision and hearing screens at 6–12 months post-diagnosis.

**Case 1**

One week after undergoing autologous stem cell transplantation for a chemotherapy-refractory haematological malignancy, a 67-year-old man developed fevers and confusion. There was no associated headache, nausea, vomiting, photophobia or neck stiffness. He was newly disoriented to time, place and person, and bedbound with increased tone in all four limbs, bilateral extensor plantar responses and pathologically brisk reflexes. Funduscopic examination showed normal optic discs, but absent spontaneous venous pulsations.

The differential diagnosis was felt to include systemic illness with delirium; infective encephalitis and disease progression with new CNS involvement. LP was attempted but was unsuccessful due to adverse anatomy. He was listed for interhospital transfer for CT-guided LP and started on empirical CNS dose meropenem and aciclovir. Contrast-enhanced MRI brain was reported as showing new leptomeningeal enhancement, concerning for disease progression with new CNS involvement.

Despite difficulties obtaining CSF, serum CrAg was not sent. Unfortunately, the patient continued to deteriorate and repeat imaging demonstrated worsening hydrocephalus. On expert review in a Neuroradiology multidisciplinary team meeting, his MRI was rereported as showing signs consistent with fungal meningitis with emerging hydrocephalus. His case was discussed with the local neurosurgical team who performed same-day programmable VP shunt insertion. Perioperative CSF was CrAg positive and there was subsequent light growth of C. neoformans on fungal culture. He was started on IV LAmB and flucytosine and made an excellent recovery. He has now transitioned to oral fluconazole and is due to undergo elective VPS removal as there was no clinical or radiologic deterioration after his shunt was effectively ‘turned off’. ^38^

Learning points/questions arising from this case:

- Was this de novo CM following transplantation or unmasking of underlying disease following engraftment in a patient previously on long-term chemotherapy?
- Should we be routinely testing for (serum) CrAg as part of the workup for allogeneic stem cell and solid organ transplantation?
- Confirms importance of testing for serum CrAg in immunosuppressed patients in whom LP proves unsuccessful or is contraindicated.
- This patient had a non-specific clinical presentation without headache and meningism—clinicians must maintain a high index of suspicion for CM.
- Importance of a strong, functional multidisciplinary team.
- Importance of knowledge of neuroimaging findings in CM.

**Case 2**

A 23-year-old woman presented to an urban specialist referral hospital in Western Uganda with a two week history of severe headache, vomiting, visual and hearing impairment, low-grade fevers and recurrent generalised tonic clonic seizures. She also reported a 4-week history of night sweats and a productive cough. She had been diagnosed with HIV several years previously but reported good adherence to antiretroviral therapy for the past 3 years. Her GCS was 15/15. Neurological examination identified right sixth nerve palsy with bilateral pupillary dilatation and reduced visual acuity.

Bedside finger-prick serum CrAg was positive. After blood tests confirmed normal platelet count and clotting function, she proceeded directly to lumbar puncture (LP). CSF analysis revealed low opening pressure (7 cmH_2_O), lymphocytic pleocytosis (WCC 16 cells/mm^3^, 78% lymphocytes), elevated protein (10 mg/dL) and normal glucose (CSF glucose 4.6 mmol/l, blood glucose 6 mmol/l). Bedside CSF CrAg was positive. She was subsequently found to have a serum HIV viral load of 3302 copies/mL and a low CD4 count of 6 cells/µL. Sputum GeneXpert testing was negative, and a plain chest radiograph unremarkable, effectively excluding concurrent disseminated tuberculosis infection.

She was diagnosed with cryptococcal meningitis and treated with 7 days intravenous amphotericin B deoxycholate and oral flucytosine, followed by 7 days of high-dose oral fluconazole. She underwent scheduled therapeutic LP at day 3 of hospitalisation, as per local protocols, which identified a normal opening pressure of 19 cmH_2_O. Her symptoms initially improved with treatment, but on day 6 of her hospital stay, she developed rebound headaches, vomiting and recurrent seizures. Repeat therapeutic LP revealed a newly raised opening pressure of 37 cmH_2_O and she had 30 ml CSF drained. Repeat, contrast-enhanced CT head on day 14 of hospitalisation showed ventricular dilatation, but no cryptococcomas. She underwent 74 therapeutic lumbar punctures, guided by her symptoms, over the course of a 46 day hospital stay. She had a good neurological outcome, with marked improvement in her visual acuity and resolution of her raised ICP symptoms and transitioned successfully to consolidation therapy with further follow-up in local HIV clinic.^63^

Learning points arising from this case:

- This case demonstrates the utility of (bedside) fingerprick serum CrAg testing, which gave an immediate clue to the diagnosis.
- Do not forget about other opportunistic infections in patients with advanced HIV (her symptoms were also compatible with a diagnosis of disseminated tuberculosis).
- Patients can develop raised ICP even with normal baseline LP OP and when established on treatment—need to maintain close follow-up and ideally have a clear schedule for LPs.
- This case illustrates marked global health inequalities in cryptococcal meningitis management; despite persistently raised opening pressures on multiple lumbar punctures and ventriculomegaly on neuroimaging (which would almost certainly have led neurosurgeons to intervene in a high-income setting), she had to endure prolonged, distressing symptoms, repeated procedures and a long hospital stay.

## Conclusion

**Figure 8:**
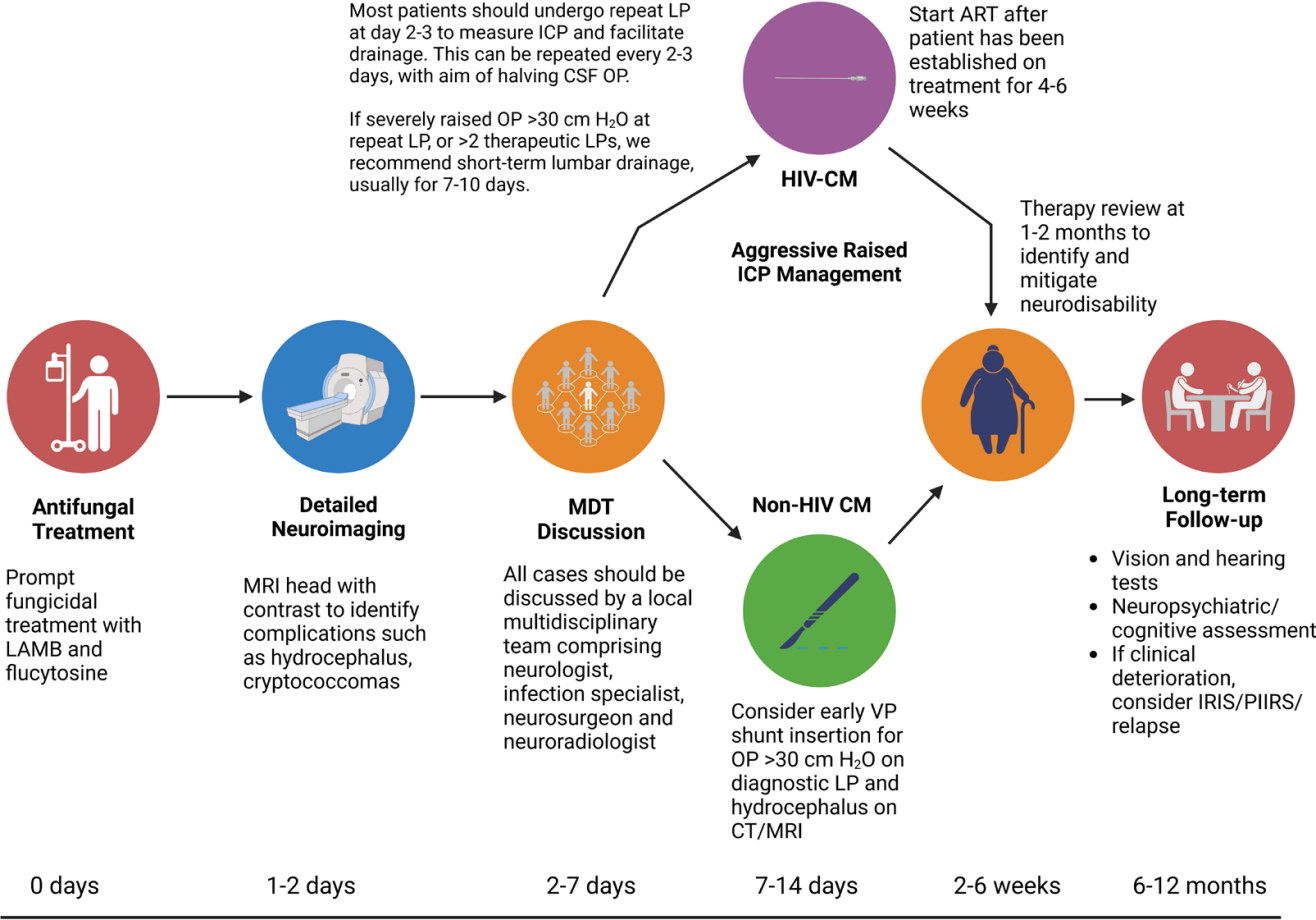
Cryptococcal meningitis (CM) management workflow. We suggest a gold-standard approach to managing an adult with CM as might be achieved in a high-income setting, with associated timeline. Core principles of management are prompt, aggressive intravenous antifungal treatment; aggressive raised ICP management; early, detailed neuroimaging to identify complications and guide MDT discussions and long-term follow-up focusing on common sequelae such as neurocognitive impairment. CSF, cerebrospinal fluid; ICP, intracranial pressure; LAMB, liposomal amphotericin B; MDT, multidisciplinary team.

While most cases of CM continue to occur in people living with HIV in Africa, CM is an important diagnosis not to miss in HIV-negative individuals with other forms of immunosuppression, and immunocompetent hosts with lymphocytic meningitis, particularly in the presence of raised opening pressure. CM carries high morbidity and mortality, particularly in HIV-negative patients, which reflects diagnostic delays and inadequate ICP management. There are divergent disease phenotypes, treatment paradigms and complications in people living with HIV, solid organ transplant recipients and NHNT patients. We propose a practical approach to the diagnosis and management of CM, recognising the differences between these groups (figure 8). Neurologists should reframe their conception of CM to encapsulate this clinical heterogeneity. CrAg testing is cheap and rapid, with a high negative predictive value in symptomatic patients; clinicians should apply this more liberally in future, notably in the assessment of immunosuppressed patients presenting with new neurology alongside people living with advanced HIV with CD4 counts below 200 cells/µL. Routine MDT discussions for these complex cases, involving neurology, infectious diseases and neurosurgical specialists, is likely to hold the key to improving long-term outcomes in all settings.

**Key points**

- The scope of cryptococcal meningitis (CM) extends beyond HIV—the main error leading to diagnostic delays and poor outcomes is clinicians’ failure to consider CM in HIV-negative patients.
- CM carries high morbidity and mortality, particularly in HIV-negative patients, which reflects diagnostic delays and inadequate ICP management.
- Cryptococcal antigen testing is cheap and rapid, with a high negative predictive value in symptomatic patients.
- Contrast-enhanced neuroimaging (MRI where available) is a useful adjunct to microbiological tests, which helps to identify complications of CM that change management.
- Raised ICP is an important complication of CM, associated with increased morbidity and mortality; serial therapeutic lumbar puncture or neurosurgical involvement is often necessary.
- A multidisciplinary team approach helps to ensure that patients receive optimal immediate and long-term treatment and appropriate aftercare including disability management.

**Further reading**

- **A -** May RC, Stone NRH, Wiesner DL, Bicanic T, Nielsen K. Cryptococcus: From environmental saprophyte to global pathogen. Vol. 14, Nature Reviews Microbiology. Nature Publishing Group; 2016. p. 106–17.
- **B-** Jarvis JN, Lawrence DS, Meya DB, Kagimu E, Kasibante J, Mpoza E, *et al*. Single-dose liposomal amphotericin B treatment for cryptococcal meningitis. New Engl.J Med 2022;386:1009–1120 doi: 10.1056/NEJMoa2111904
- **C -** Chang CC, Harrison TS, Bicanic TA, Chayakulkeeree M, Sorrell TC, Warris A, *et al*. Global guideline for the diagnosis and management of cryptococcosis: an initiative of the ECMM and ISHAM in cooperation with the ASM. Lancet Infect Dis (Internet). 2024 Apr 6; Available from: https://doi.org/10.1016/S1473-3099(23)00731-4

## supplementary material

10.1136/pn-2024-004133 online supplemental file 1

## Data Availability

No data are available.
