# Supplementary material for: Reframing the clinical phenotype and management of cryptococcal meningitis
Source: Pract Neurol. 2024 Jul 12;25(1):e004133. doi: 10.1136/pn-2024-004133 (PMC11877062; doi:10.1136/pn-2024-004133)
Supplement: online supplemental file 1 [file pn-25-1-s001.pdf]

Online Supplemental Material 1

Medical Management of Cryptococcal Meningitis

Liposomal amphotericin B (LAmB) sequesters active amphotericin B in the fungal cell membrane, reducing interaction with human cells, and thus adverse effect frequency. Where available, this is preferred over amphotericin B deoxycholate. Historically, HIV-CM cases have received two weeks of amphotericin B at induction (Perfect *et al.*, 2010), but this is costly, entails prolonged hospital stays, and, in low-income settings, LAmB can be difficult to source, owing to competing indications for its use, such as visceral leishmaniasis. The 2018 multi-centre ACTA trial showed that both a shortened 1-week IV amphotericin B regimen (alongside 2 weeks of oral flucytosine), and a dual oral regimen (2 weeks of fluconazole and flucytosine), were non-inferior to a 2-week IV amphotericin B/flucytosine induction (Molloy *et al.*, 2018). While this provided evidence for efficacy of cheaper drug regimens in hyperendemic sub-Saharan African settings, even these simplified combinations remained out-of-reach for many patients. However, this is set to change, in light of the 2022 AMBITION-cm trial. AMBITION-cm demonstrated fewer adverse effects and non-inferior 10-week mortality amongst adults who received a single, high-dose of LAmB (10 mg/kg), followed by high-dose fluconazole (1200 mg OD) therapy, and a 2-week course of flucytosine 25 mg/kg QDS *versus* the 1-week previous WHO-recommended regimen (LAmB 1 mg/kg and flucytosine 25 mg/kg for 1 week, followed by fluconazole 1200 mg OD monotherapy during the second week). This large, robust trial involved 844 patients across several low-income countries (Jarvis *et al.*, 2022), and the novel regimen has now been adopted by the WHO for use amongst HIV-CM patients in low-resource settings.

This may translate to HIV-CM management in high-income settings, but further work is needed to determine if it is appropriate for SOT and NHNT groups, who currently receive longer induction courses. Of note, the control arm in AMBITION-cm received an induction regimen which is not standard for PLHIV, SOT and NHNT groups in high-income settings—such patients usually receive a 2-week course of LAmB 3-4 mg/kg plus flucytosine 25 mg/kg QDS, and a lower fluconazole consolidation dose of 400-800 mg OD. There are particular concerns about the use of high-dose fluconazole therapy in high-income settings, due to risks of drug interactions in patients who are generally older and more comorbid than those enrolled on AMBITION-cm, and the lower tolerance of dose-dependent azole hepatotoxicity amongst clinicians working in these settings. For these reasons, recently published consensus global guidelines suggest that patients in low-income settings should be treated with the novel regimen trialled in AMBITION-cm, while those in high-income settings should continue to receive a two-week high-dose LAmB/flucytosine induction therapy (Chang *et al.*, 2024). Special groups for whom prolonged 4-6 week induction therapy should be considered include patients with cryptococcomas, and/or confirmed *C. gattii* non-HIV CM.

Fluconazole consolidation therapy, at a dose of 400-800 mg OD, is recommended for all patients, in all settings, for a duration of 8 weeks. Where possible, the transition to consolidation therapy should be informed by repeat CSF sampling and culture at day 14. The presence of yeast cells on microscopy alone (with no growth on culture), and persistently positive CrAg, should not prompt diversion from the

usual antifungal regimen. However, persistent *Cryptococcus* growth implies viable cells, and should prompt more prolonged fungicidal LAmB-based induction.

The end of the consolidation therapy period represents an excellent juncture for clinical review. Ongoing symptoms may reflect microbiological relapse, due to suboptimal fluconazole adherence, drug resistance, or reduced efficacy due to drug interactions; alternative CNS pathology, particularly in patients with profound immunosuppression; and paradoxical inflammatory treatment reactions. Symptomatic patients should undergo repeat neuroimaging, and lumbar puncture with manometry and CSF fungal culture. Culture positivity indicates microbiological relapse, and should prompt re-induction, and antifungal susceptibility testing. The utility of other azole drugs in CM consolidation and maintenance therapy has not been assessed in large trials, and these drugs remain out-of-reach in low- and middle-income countries. They should also be used cautiously in solid-organ transplant recipients, given important interactions with tacrolimus and ciclosporin. Nonetheless, their use may be reasonable in patients with strains of *Cryptococcus* with emerging/confirmed resistance to fluconazole, or significant drug interactions. Culture-negative patients, with no identifiable alternative diagnosis, are likely to have a paradoxical inflammatory treatment reaction, discussed at length in the Complications section in the main article. Asymptomatic patients can transition to lower dose maintenance fluconazole 200 mg OD. This is continued for PLHIV until HIV RNA levels are undetectable and CD4 count has increased to >100/μl; and for 12 months in non-HIV CM.

In low-resource settings, suboptimal treatment variations may be necessary, owing to limited drug availability and/or prohibitive costs. CM is universally fatal if untreated, with 60-70% of patients dying within 10 weeks of diagnosis. Fluconazole monotherapy, fluconazole/flucytosine combination therapy, and amphotericin/flucytosine combination therapy reduce 10-week mortality to 50%, 35% and 25%, respectively (Shiri *et al.*, 2020). We commend recent global consensus guidelines to readers, which contain useful Figures outlining optimal drug combinations for CM management in low-resource settings (Chang *et al.*, 2024).

Adjuncts, including sertraline and mannitol, confer no prognostic benefit (Hu *et al.*, 2017; Rhein *et al.*, 2019). Steroids do not improve mortality, and may, in fact, be harmful (Beardsley *et al.*, 2016). However, positive outcomes have been reported with steroids in specific subgroups, notably patients with paradoxical inflammatory treatment reactions, and cryptococcomas with peri-lesional oedema and mass effect, and guidelines support their use in these circumstances. The preferred treatment regimen for cryptococcal antigenaemia with negative CSF CrAg, blood cultures, and no extracranial cryptococcosis manifestations, is 6-12 months of oral fluconazole monotherapy (Chang *et al.*, 2024).

CM is rare in pregnancy, but poses substantial risks to mother and child, which must be balanced against teratogenic effects of antifungal treatment. Treatment regimens are based on expert opinion rather than high-quality trial-based evidence. Prolonged amphotericin B monotherapy has been used to good effect in both cryptococcal antigenaemia and CM in pregnancy (Bright *et al.*, 2018; Pastick *et al.*, 2020). Despite this, mothers should be warned of risks of poor fetal outcomes, which may reflect wider epiphenomena of poorly controlled HIV, rather than sequelae of cryptococcosis

per se. Flucytosine should be given only on expert advice, when benefits are felt to outweigh risks. Fluconazole is teratogenic in animal models, and, while it is often used in practice during pregnancy in low-income settings, it should be avoided altogether whenever possible. Clinicians should warn women living with HIV on fluconazole prophylaxis about its teratogenicity, and advocate for robust contraception use. However, fluconazole is safe during breast-feeding, and we advise early switch to fluconazole monotherapy in the post-partum period (Kaplan *et al.*, 2015).

References

Beardsley J, Wolbers M, Kibengo FM, Ggayi AB, Kamali A, Cuc NT, Binh TQ, Chau NV, Farrar J, Merson L, Phuong L, Thwaites G, Van Kinh N, Thuy PT, Chierakul W, Siriboon S, Thiansukhon E, Onsanit S, Supphamongkholchaikul W, Chan AK, Heyderman R, Mwinjiwa E, van Oosterhout JJ, Imran D, Basri H, Mayxay M, Dance D, Phimmasone P, Rattanavong S, Lalloo DG, Day JN & CryptoDex I. (2016). Adjunctive Dexamethasone in HIV-Associated Cryptococcal Meningitis. *N Engl J Med* **374,** 542-554.

Bright PD, Lupiya D, van Oosterhout JJ, Chen A, Harrison TS & Chan AK. (2018). The treatment of a pregnant HIV positive patient with cryptococcal meningitis in Malawi. Case report and review of treatment options. *Med Mycol Case Rep* **19,** 9-12.

Chang CC, Harrison TS, Bicanic TA, Chayakulkeeree M, Sorrell TC, Warris A, Hagen F, Spec A, Oladele R, Govender NP, Chen SC, Mody CH, Groll AH, Chen YC, Lionakis MS, Alanio A, Castaneda E, Lizarazo J, Vidal JE, Takazono T, Hoenigl M, Alffenaar JW, Gangneux JP, Soman R, Zhu LP, Bonifaz A, Jarvis JN, Day JN, Klimko N, Salmanton-Garcia J, Jouvion G, Meya DB, Lawrence D, Rahn S, Bongomin F, McMullan BJ, Sprute R, Nyazika TK, Beardsley J, Carlesse F, Heath CH, Ayanlowo OO, Mashedi OM, Queiroz-Telles Filho F, Hosseinipour MC, Patel AK, Temfack E, Singh N, Cornely OA, Boulware DR, Lortholary O, Pappas PG & Perfect JR. (2024). Global guideline for the diagnosis and management of cryptococcosis: an initiative of the ECMM and ISHAM in cooperation with the ASM. *Lancet Infect Dis*.

Hu Z, Yang Y, Cheng J, Cheng C, Chi Y & Wei H. (2017). The use of mannitol in HIV-infected patients with symptomatic cryptococcal meningitis. *Drug Discov Ther* **10,** 329-333.

Jarvis JN, Lawrence DS, Meya DB, Kagimu E, Kasibante J, Mpoza E, Rutakingirwa MK, Ssebambulidde K, Tugume L, Rhein J, Boulware DR, Mwandumba HC, Moyo M, Mzinganjira H, Kanyama C, Hosseinipour MC, Chawinga C, Meintjes G, Schutz C, Comins K, Singh A, Muzoora C, Jjunju S, Nuwagira E, Mosepele M, Leeme T, Siamisang K, Ndhlovu CE, Hlupeni A, Mutata C, van Widenfelt E, Chen T, Wang D, Hope W, Boyer-Chammard T, Loyse A, Molloy SF, Youssouf N, Lortholary O, Lalloo DG, Jaffar S, Harrison TS & Ambition Study G. (2022). Single-Dose Liposomal Amphotericin B Treatment for Cryptococcal Meningitis. *N Engl J Med* **386,** 1109-1120.

Kaplan YC, Koren G, Ito S & Bozzo P. (2015). Fluconazole use during breastfeeding. *Can Fam Physician* **61,** 875-876.

Molloy SF, Kanyama C, Heyderman RS, Loyse A, Kouanfack C, Chanda D, Mfinanga S, Temfack E, Lakhi S, Lesikari S, Chan AK, Stone N, Kalata N, Karunaharan N, Gaskell K, Peirse M, Ellis J, Chawinga C, Lontsi S, Ndong JG, Bright P, Lupiya D, Chen T, Bradley J, Adams J, van der Horst C, van Oosterhout JJ, Sini V, Mapoure YN, Mwaba P, Bicanic T, Lalloo DG, Wang D, Hosseinipour MC, Lortholary O, Jaffar S, Harrison TS & Team ATS. (2018). Antifungal Combinations for Treatment of Cryptococcal Meningitis in Africa. *N Engl J Med* **378,** 1004-1017.

Pastick KA, Nalintya E, Tugume L, Ssebambulidde K, Stephens N, Evans EE, Ndyetukira JF, Nuwagira E, Skipper C, Muzoora C, Meya DB, Rhein J, Boulware DR & Rajasingham R. (2020). Cryptococcosis in pregnancy and the postpartum period: Case series and systematic review with recommendations for management. *Med Mycol* **58,** 282-292.

Perfect JR, Dismukes WE, Dromer F, Goldman DL, Graybill JR, Hamill RJ, Harrison TS, Larsen RA, Lortholary O, Nguyen MH, Pappas PG, Powderly WG, Singh N, Sobel JD & Sorrell TC. (2010). Clinical practice guidelines for the management of cryptococcal disease: 2010 update by the infectious diseases society of america. *Clin Infect Dis* **50,** 291-322.

Rhein J, Huppler Hullsiek K, Tugume L, Nuwagira E, Mpoza E, Evans EE, Kiggundu R, Pastick KA, Ssebambulidde K, Akampurira A, Williams DA, Bangdiwala AS, Abassi M, Musubire AK, Nicol MR, Muzoora C, Meya DB, Boulware DR & team A-C. (2019). Adjunctive sertraline for HIV-associated cryptococcal meningitis: a randomised, placebo-controlled, double-blind phase 3 trial. *Lancet Infect Dis* **19,** 843-851.

Shiri T, Loyse A, Mwenge L, Chen T, Lakhi S, Chanda D, Mwaba P, Molloy SF, Heyderman RS, Kanyama C, Hosseinipour MC, Kouanfack C, Temfack E, Mfinanga S, Kivuyo S, Chan AK, Jarvis JN, Lortholary O, Jaffar S, Niessen LW & Harrison TS. (2020). Addition of Flucytosine to Fluconazole for the Treatment of Cryptococcal Meningitis in Africa: A Multicountry Cost-effectiveness Analysis. *Clin Infect Dis* **70,** 26-29.
